# Morpho-Structural, Thermal and Mechanical Properties of PLA/PHB/Cellulose Biodegradable Nanocomposites Obtained by Compression Molding, Extrusion, and 3D Printing

**DOI:** 10.3390/nano10010051

**Published:** 2019-12-24

**Authors:** Adriana Nicoleta Frone, Dan Batalu, Ioana Chiulan, Madalina Oprea, Augusta Raluca Gabor, Cristian-Andi Nicolae, Valentin Raditoiu, Roxana Trusca, Denis Mihaela Panaitescu

**Affiliations:** 1 Polymer Department, National Institute for Research & Development in Chemistry and Petrochemistry ICECHIM, 202 Splaiul Independentei, 060021 Bucharest, Romania; ioana.chiulan@icechim.ro (I.C.); madalinna_09@yahoo.com (M.O.); ralucagabor@yahoo.com (A.R.G.); ca_nicolae@yahoo.com (C.-A.N.); vraditoiu@yahoo.com (V.R.); panaitescu@icechim.ro (D.M.P.); 2 Materials Science and Engineering Faculty, University Politehnica of Bucharest, 060042 Bucharest, Romania; dan_batalu@yahoo.com; 3 Science and Engineering of Oxide Materials and Nanomaterials, University Politehnica of Bucharest, 1-7 Gh. Polizu Street, 011061 Bucharest, Romania; truscaroxana@yahoo.com

**Keywords:** cellulose nanocrystals, biopolymers, reactive blending, extrusion, 3D printing, polylactic acid, morphology, dynamic mechanical analysis

## Abstract

Biodegradable blends and nanocomposites were produced from polylactic acid (PLA), poly(3-hydroxybutyrate) (PHB) and cellulose nanocrystals (NC) by a single step reactive blending process using dicumyl peroxide (DCP) as a cross-linking agent. With the aim of gaining more insight into the impact of processing methods upon the morphological, thermal and mechanical properties of these nanocomposites, three different processing techniques were employed: compression molding, extrusion, and 3D printing. The addition of DCP improved interfacial adhesion and the dispersion of NC in nanocomposites as observed by scanning electron microscopy and atomic force microscopy. The carbonyl index calculated from Fourier transform infrared spectroscopy showed increased crystallinity after DCP addition in PLA/PHB and PLA/PHB/NC, also confirmed by differential scanning calorimetry analyses. NC and DCP showed nucleating activity and favored the crystallization of PLA, increasing its crystallinity from 16% in PLA/PHB to 38% in DCP crosslinked blend and to 43% in crosslinked PLA/PHB/NC nanocomposite. The addition of DCP also influenced the melting-recrystallization processes due to the generation of lower molecular weight products with increased mobility. The thermo-mechanical characterization of uncross-linked and cross-linked PLA/PHB blends and nanocomposites showed the influence of the processing technique. Higher storage modulus values were obtained for filaments obtained by extrusion and 3D printed meshes compared to compression molded films. Similarly, the thermogravimetric analysis showed an increase of the onset degradation temperature, even with more than 10 °C for PLA/PHB blends and nanocomposites after extrusion and 3D-printing, compared with compression molding. This study shows that PLA/PHB products with enhanced interfacial adhesion, improved thermal stability, and mechanical properties can be obtained by the right choice of the processing method and conditions using NC and DCP for balancing the properties.

## 1. Introduction

The extensive use of petroleum-based plastics for industrial and consumer products along with ineffective waste management leads to serious environmental and economic issues [1,2,3]. Around 5000 million metric tons of plastic waste is accumulating in landfills or in the natural environment [4]. Only the plastic waste generated annually in coastal countries reaches 275 million metric tons, with 4.8 to 12.7 million metric tons entering the ocean [5]. In consequence, scientists are encouraged to seek for biodegradable materials derived from renewable resources which show comparable or enhanced properties to those of petroleum-based plastics.

Polylactic acid (PLA) is considered the frontrunner of biodegradable polymers since it is already used in several commercial applications as an alternative to certain petroleum-based plastics. PLA is available on the market at a similar price with that of common plastics like polypropylene [6]. Besides its excellent biocompatibility, biodegradability, renewability, and good mechanical strength, PLA has the important advantage of easy processability using common technologies for thermoplastic polymers (melt mixing, extrusion, and injection molding) or 3D printing [3,7,8].

Despite these valuable properties, PLA has a small elongation before breaking, poor impact strength and thermal resistance, low heat distortion temperature and rate of crystallization, like most bio-based materials. To solve these problems, many approaches were explored, such as the addition of fillers and nucleating agents, copolymerization, or melt blending [9,10,11,12]. Blending PLA with poly (3-hydroxybutyrate) (PHB) is often used to improve some properties, based on their similar melting temperature and high crystallinity of PHB [11,12]. Still, PLA-PHB polymer blends are immiscible and, therefore, compatibilization methods should be used to obtain better properties [13]. Peroxide induced cross-linking is a frequently used technique to generate high-performance polymeric blends. Thus, an improvement of properties can be achieved by in situ reactive compatibilization, which is an effective, fast, solvent-free, low-cost and ecologic method to process PLA blends [14]. The decomposition of peroxides into free radicals promotes cross-linking, chain scission and branching in the polymer matrix thus influencing the melting behavior, crystallinity, and mechanical properties [15]. Dicumyl peroxide (DCP) was used in previous works to compatibilize PLA polymeric blends. PLA/poly(butylene adipate-co-terephthalate) was compatibilized using 0.2% DCP, leading to improved tensile strength, ductility and impact strength [16]. An increase of the elongation at break with 140% was obtained in the case of PLA/polycaprolactone blends containing small amounts of DCP [17]. Similarly, PHB/PLA blends with increased interfacial adhesion and improved mechanical properties were obtained by DCP cross-linking [18]. However, a higher concentration of DCP in these blends increased the cross-link density and decreased the melting and glass transition temperatures along with crystallinity [19].

Further improvement of biopolymers properties is usually necessary to compete with common synthetic polymers and the addition of fillers represents a verified method to enhance their mechanical properties [20,21]. Cellulose nanofibers or nanocrystals and microcrystalline cellulose were widely utilized for the reinforcement of polymeric blends [22,23,24]. Among them, cellulose nanocrystals with their rod-like morphology, high hydroxyl functionality, and surface area, are considered a very promising green reinforcing agent, which can impart targeted properties to the polymeric matrix [3,10,25,26]. Thus, cellulose nanocrystals functionalized with surfactants were incorporated in PLA-PHB composites, by melt mixing in a micro extruder, improving the mechanical properties of the blend [11,27]. However, PLA—poly(3-hydroxybutyrate-co-3-hydroxyvalerate) (PHBV) reinforced with TEMPO-oxidized NC using a solvent casting technique showed improved properties compared to the polymer blend only at an optimal NC concentration [28].

The processing technique and parameters, as well as the additives, could strongly influence the efficiency of the reinforcing or cross-linking agents and the thermal and mechanical properties of final biomaterials. Zheng et al. directly compared solvent casting and melt processing approaches in the case of PHBV-CNC composites and concluded that melt processing did not produce composites with the same level of dispersion and mechanical properties as solvent-casting, even when the CNCs were coated with polyethylene glycol to prevent particle agglomeration [29]. However, solution casting may be used for small production and the use of solvents may impact the environment and human health [30]. Besides, melt compounding is the most popular technique to obtain blends or composites in industry and more attempts to incorporate NC in PLA using melt mixing or extrusion methods were reported in the last years [31,32]. For example, NC as water suspension was firstly premixed with PHB and the masterbatch was further melt mixed with PLA, ensuring a better dispersion of the filler [32].

3D printing technique was also used to manufacture PLA blends [8]. 3D printing is a novel technique that allows the rapid fabrication of a physical prototype from a virtual concept using a three-dimensional computer-aided design (CAD). This approach allows the reducing of manufacturing time up to 50% even if the model complexity is very high [33,34]. Among the rapid prototyping technologies, the most frequently used is fused deposition modeling (FDM). FDM is based on the heating of a thermoplastic filament up to its melting point and the extrusion of the material followed by layer by layer deposition on a substrate to create a three-dimensional model [35,36]. Ausejo et al. [37] studied the influence of 3D printing conditions (horizontal or vertical direction, contact time) and specimen size on the crystallinity, thermal and mechanical properties of PLA and PLA/polyhydroxyalkanoate (PHA) blends. A drawback in the 3D printing process is represented by the lack of strength and thermal stability when dealing with biopolymers. Biopolymers are kept for the entire printing period at the melting temperature and they should remain stable throughout the printing process without changing their characteristics [38]. Mencík et al. tried to improve the 3D printing behavior of PHB/PLA (70/30) blends by using different plasticizers [39]. Although PLA and PLA/PHA blends have been extensively studied for additive manufacturing, the literature on PLA/PHB/NC biocomposites for 3D printing is scarce. Moreover, no comparative study on the influence of the processing techniques including melt compounding, compression molding, extrusion and 3D printing on the properties of PLA based materials was found in the literature.

Therefore, in this work cellulose nanocrystals were incorporated into PLA/PHB blends by a single step reactive blending process using DCP as a cross-linking agent. Three different processing methods, namely, compression molding, extrusion, and 3D printing were used for this purpose and their influence on the morphological, thermal and mechanical properties of PLA/PHB/NC biocomposite cross-linked with DCP was studied for the first time. The processing methods were chosen to involve increasing temperature and shear stresses to the material in the following order compression molding < extrusion < 3D printing. Moreover, the cellulose nanofibers used as a reinforcing agent in PLA/PHB blends were isolated from agricultural residues, which could increase the final value and benefits of the resulted materials. Plum shells are an agricultural residue which is discharged through incineration or, most often, disposed of in the environment as garbage, since they have no industrial usage, thus representing an environmental problem. Using them as a source of high-value NC represents a sustainable concept from both environmental and economic perspectives.

## 2. Materials and Methods

### 2.1. Materials

Plum seed shells derived from Romanian varieties of plum trees were used as raw material to produce cellulose nanocrystals (NC). Sodium hydroxide (NaOH, ≥99%, Carl Roth, Karlsruhe, Germany), sulfuric acid (H_2_SO_4_, 95–97%, Fluka, Buchs, Switzerland), sodium chlorite (NaClO_2_, 80%, Carl Roth, Karlsruhe, Germany), hydrogen peroxide (H_2_O_2_, 30%, Merck, Darmstadt, Germany) and acetic acid (CH_3_COOH, 99–100% min., Carl Roth, Karlsruhe, Germany) were used for cellulose nanocrystals production. Amorphous grade polylactic acid pellets (PLA, Ingeo™ biopolymer 4043D, 1.24 g cm^−3^), L-lactide content about 98% from NatureWorks (Blair, NE, USA), and pelletized PHB from Goodfellow (Huntingdon, UK, 1.25 g cm^−3^) were used as polymer matrices. Cellulose nanocrystals, with a diameter between 30 and 80 nm, were isolated from plum seed shells and were used to modify the PLA/PHB blend. Dicumyl peroxide (DCP, 98%, Sigma-Aldrich, Steinheim, Germany) was used as a cross-linker.

### 2.2. Isolation of Cellulose Nanocrystals from Plum Seed Shells

The plum seed shells were ground and then sieved to achieve the particle size of 0.16 mm. The obtained powder was subjected to Soxhlet extraction using a mixture of ethanol:water (80:20) for 2 h at 130°C in order to remove any impurities. The resulted solid mass was pretreated through soda pulping method (8 wt% NaOH at 80 °C for 2 h—the procedure was repeated four times) followed by bleaching (1.5 wt% NaClO_2_ treatment at 70 °C for 5 h—the procedure was repeated three times) to remove the hemicellulose and lignin impurities. Hydrogen peroxide was also employed at the final bleaching step (4 wt% H_2_O_2_ at 60 °C for 6 h). Acid hydrolysis of the bleached pulp was carried out with 60 wt% H_2_SO_4_ at 40 °C for 150 min. The hydrolysis reaction was stopped by adding chilled deionized water followed by centrifugation (~7000 rpm) to remove the excess acid. The NC suspension was dialyzed with distilled water using regenerated cellulose membrane (cut off molecular weight 6000–8000 Da, Carl Roth Spectra/Por 1) until a final pH of ~7 was attained. Ultrasonic treatment was also carried out in an ultrasonic bath Elmasonic S 15 H (Singen, Germany) for 30 min in an ice bath to maintain the temperature below 30°C. Subsequently, the final suspension was lyophilized for 72 h at −84 °C to obtain the freeze-dried NCs.

### 2.3. Preparation of PLA/PHB/NC Nanocomposites

PLA and PHB pellets were dried in a vacuum oven at 80 °C for 4h prior to melt-blending. PLA/PHB (75:25 proportion) blends containing 1wt% NC were prepared in a Brabender Plasticorder LabStation (Duisburg, Germany) equipped with a 30 cm^3^ cell at a temperature of 175°C for 8 min at a rotor speed of 60 rpm. NC was added after the complete melting of polymers and DCP was incorporated prior to NC addition in the case of reactive melt-blended samples. One part of the blended materials was molded on a laboratory two-roll-mill heated to 80 °C for ~30 s and compression molded into sheets of 0.3 mm in thickness, using an electrically heated press (P200E, Dr. Collin, Ebersberg, Germany) at 170 °C with 150 s of preheating at 0.5 MPa and 60 s of compression at 10 MPa. The plates were cooled in a cooling cassette and allowed to sit at room temperature for four weeks before characterization. PLA/PHB sample was processed under the same conditions and was used as a reference.

The other part of the blend was further used for the extrusion of filaments with 1.75 mm ± 0.05 from both uncross-linked and cross-linked PLA/PHB/NC nanocomposites using a co-rotating twin-screw extruder type DSE 20 Brabender (Duisburg, Germany) at a screw rate of 60 rpm. The extruder has six independent temperature control zones along the barrel length which were set as following: first at 160 °C, the next two at 165 °C, and the last three at 170 °C. The extruded filaments were cooled down with blown air, manually rolled on a drum and further used for printing 3D meshes. A neat PLA/PHB filament was obtained in the same conditions and served as a control sample.

Mesh prototypes (5 cm × 5 cm, with voids of 5 mm × 5mm, and 0.5 mm thickness) were designed with Autodesk Inventor Professional 2020, sliced with Simplify 3D 4.1, and printed with WASP Delta 2040 Turbo 2 printer (Massa Lombarda, Italy) using the following parameters: nozzle diameter of 0.4 mm, nozzle melting temperature of 200 °C, printing speed: 2000 mm min^−3^. All the specimens were printed directly on the heated bed (60 °C) without any supports.

**Figure 1:**
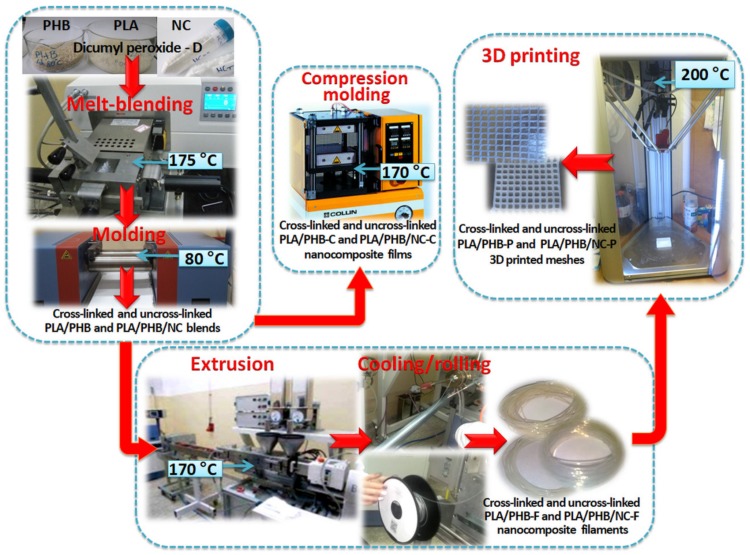
Schematic representation of PLA/PHB blends and nanocomposites preparation and processing of films, filaments, and 3D-printed meshes.

The formulations of the obtained nanocomposites, their notations according to the processing method are given in Table 1 while Figure 1 illustrates the processing methods used in this paper.

### 2.4. Characterization Methods

A Tecnai G2 F20 Twin Cryo-Tem transmission electron microscope (TEM) (FEI, Hillsboro, OR, USA), operating at 120 kV, was used to observe the morphological features of cellulose nanocrystals. Cellulose nanocrystals were examined directly, without staining for contrast enhancement. One droplet of NC suspension was deposited on carbon-coated copper grids and allowed to dry at 25 °C before observations.

The morphology of the cryo-fractured PLA/PHB nanocomposites films and filaments was studied using an FEI Quanta Inspect FEG Scanning Electron Microscope (SEM) (FEI, Hillsboro, OR, USA) at an accelerating voltage of 30 kV with a resolution of 1.2 nm. Samples were previously sputtered with gold for 30 s before the examination.

Atomic force microscopy (AFM)(MultiMode 8, Bruker, Santa Barbara, CA, USA) analysis was performed to better observe the surface dispersion of NC in the polymer matrix. The investigations were performed in Peak Force (PF) Quantitative Nanomechanical Mapping (QNM) mode, at room temperature, with a scan rate of 1 Hz and a scan angle of 90°. Etched silicon tips (nominal radius 8 nm) with a cantilever length of 225 μm and a resonance frequency of about 75 kHz were used for the measurements. The image processing and the data analysis were made with NanoScope software version 1.20.

Fourier transformed infrared (FTIR) measurements of the nanocomposite films were carried out in attenuated total reflectance (ATR) spectra in the 4000–400 cm^−1^ region using a Jasco FTIR 6300 spectrometer (Jasco Co., Tokyo, Japan) equipped with an ATR Specac Golden Gate (KRS5 lens) in order to evaluate the effect of peroxide on the chemical structure. ATR measurements were the average of 32 scans at a resolution of 4 cm^−1^.

Dynamic thermogravimetric analysis (TGA) tests were conducted by means of a TGA Q500 thermal analyzer (TA instruments, New Castle, DE, USA). Nanocomposite films, filaments and 3D-printed meshes with an average weight of about 10 mg were subjected to a heating program from room temperature up to 700 °C at 10 °C min^−1^ under nitrogen atmosphere (40 mL min^−1^).

Thermal transitions of nanocomposite films were obtained by differential scanning calorimetry measurements (DSC, Q2000, TA instruments, New Castle, DE, USA) under helium flow (25 mL min^−1^). Samples weighing around 6 mg were put in standard aluminium pans and subjected to heating/cooling cycles as follows: (i) cooling from room temperature to −45°C with 50°C min^−1^, isothermal for 3 min to delete the thermal history; (ii) heating to 200 °C with 10°C min^−1^, isothermal for 2 min; (iii) cooling to −45°C, with 10°C min^−1^, isothermal for 2 min. and (iv) heating with 10°C min^−1^ to 200 °C. The melting temperature (*T_m_*) was taken as the peak temperature of the melting endotherm. The percent of crystallinity of PLA and PHB phases in the PLA/PHB blends and nanocomposites (*X_PLA_* and *X_PHB_*), were calculated from the first heating run according to Equations (1) and (2):

(1) $$X_{PLA}\%=\frac{∆H_{m}-∆H_{cc}}{∆H_{m}^{0}\times w_{PLA}}\times 100,$$

(2) $$X_{PHB}\%=\frac{∆H_{m}}{∆H_{m}^{0}\times w_{PHB}}\times 100\;,$$

where Δ*H_m_* is the total melting enthalpy, Δ*H_cc_* is the cold crystallization enthalpy of PLA, *w_PLA_* and w_PHB_ correspond to the weight fractions of PLA and PHB in the samples while Δ*H_m_^0^* represents the theoretical melt enthalpy of a fully crystalline PLA (93.0 J g^−1^) and PHB (146 J g^−1^) [40].

The thermo-mechanical properties of nanocomposite films, filaments and 3D-printed parts were analyzed using a dynamic mechanical analyzer DMA Q800 (TA Instruments, New Castle, DE, USA) operating in multi-frequency-strain mode at a heating rate of 3 °C min^−1^. Samples with different geometries were used in DMA analysis depending on the processing method: rectangular film (12 mm × 6.9 mm × 0.3 mm) for compression molding, cylindrical (12 mm × Ø1.75 mm) for extruded filaments and filament-like geometry (12 mm × Ø1.2 mm) for printed meshes obtained after cutting the sides of the grid. Samples were equilibrated at −45 °C, kept isothermal at that temperature for 2 min, then heated to 145 °C.

## 3. Results and Discussion

### 3.1. Morphology of Cellulose Nanocrystals

**Figure 2:**
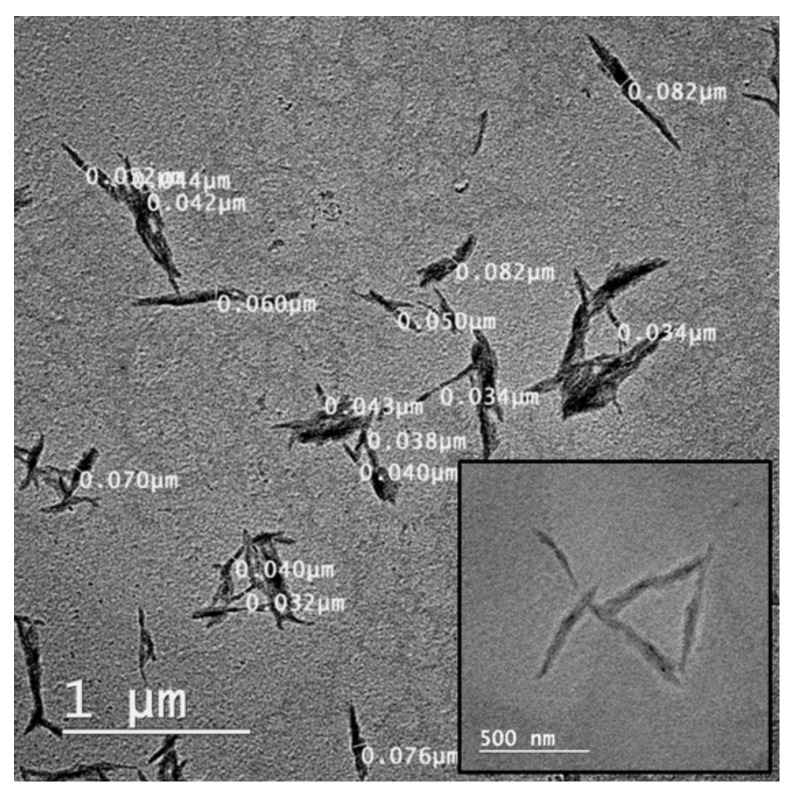
TEM images of cellulose nanocrystalsisolated from plum seed shells.

TEM images of obtained cellulose nanocrystals are given in Figure 2. NC appears as crystals with dimensions ranging from 500 to 600 nm in length and 34 to 82 nm in width.

### 3.2. Morphology of Nanocomposites as Films and Filaments

**Figure 3:**
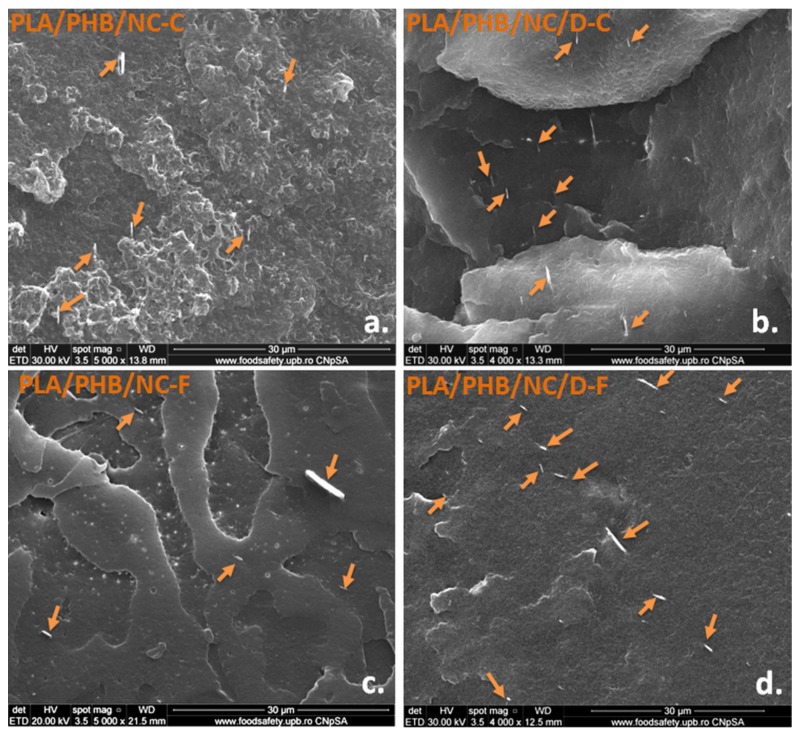
SEM images of cryo-fractured surface of the obtained nanocomposites as films (**a**,**b**) and filaments (**c**,**d**).

Morphological aspects of the cryo-fractured cross-sections of PLA/PHB nanocomposites as films and filaments were investigated by SEM and the images are shown in Figure 3a–d. PLA and PHB are semi-crystalline polymers. PHB has a higher crystallinity and can act as a nucleating agent to induce PLA crystallization into a more ordered crystalline structure [40]. PLA/PHB/NC-C film shows an irregular fractured surface with PHB crystalline domains embedded in an amorphous PLA continuous phase (Figure 3a) due to the partial compatibility of PLA and PHB [13,41]. PHB domains appear dispersed as crystalline aggregates showing poor miscibility between the two phases (Figure 3a). Voids were also observed in the SEM image of the PLA/PHB/NC-C sample, supporting the partial miscibility between the two polymers (Figure 3a).

A different morphology was observed in the PLA/PHB/NC-C film fracture surface in the presence of DCP (Figure 3b). The micrographs of fractured surfaces indicated improved miscibility of polymers, and no voids occurred. The addition of DCP had a positive effect on NC dispersion and on improving the interfacial adhesion between PLA and PHB phases, which is attributed to the development of a cross-linked network at the interface (Figure 3b, arrows). Dong et al. [18] also showed that the cross-linking of PLA/PHB blend with DCP resulted in significantly enhanced interfacial adhesion between PLA and PHB phases.

SEM images of the fractured surface of PLA/PHB-based nanocomposites filaments showed a distinct morphology when compared to that of the films due to the orientation effect in the extrusion process (Figure 3c,d). Usually, the alignment of the crystalline structure in the polymers is parallel to the extrusion direction resulting in a different structure compared to that obtained by compression [42]. Reprocessing of composites by extrusion contributed to an advanced blending of components thus resulting in a more homogenous material. As seen in Figure 3c, the fracture surface of PLA/PHB/NC-F composite filament appears rougher, flake-like morphology and the fractures were developed in different directions indicating a ductile fracture. The fracture surface of the cross-linked PLA/PHB/NC/D-F sample exhibited, as in the case of the films, a more homogenous structure and a better dispersion and adhesion of NC (Figure 3d, arrows), because of the positive effect of cross-linking agent. The NC fibers are well embedded in the PLA/PHB matrix. These observations highlight the positive effect of DCP in enhancing interfacial adhesion between NC and PLA/PHB matrix and simultaneously in improving the compatibility between PLA and PHB. Both cross-linked and uncross-linked PLA/PHB/NC-F filaments displayed plastic deformation during fracture and different fracture directions which require more energy at break, therefore, the filaments should induce increased toughness.

Cellulose nanocrystals dispersion at nano-scale and the influence of DCP are better illustrated by AFM 3D-analysis of nanocomposite films surface. The roughness of PLA/PHB nanocomposites was also examined by AFM considering that both the cross-linking agent and the NC can induce changes in the surface characteristics of the polymeric matrix. The root-mean-square roughness (RMSR), used to express the surface roughness of different materials, was calculated for both neat PLA/PHB and nanocomposites, using the NanoScope AFM software. At least five AFM topographic images were analyzed for each sample. The original AFM data were leveled by mean plane subtraction before roughness calculation, without further correction of tip dilation effect and the RMSR results were the arithmetic average of 5 images of 5 µm × 5 µm.

**Figure 4:**
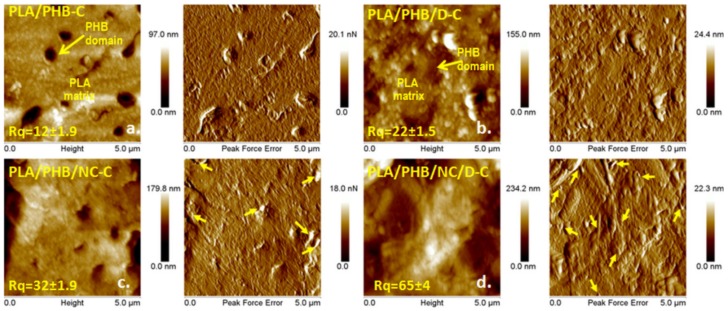
Height and peak force error images of the surface of PLA/PHB blends (**a**,**b**) and nanocomposite films (**c**,**d**), scan area of 5 µm × 5 µm; Root-mean-square roughness values added on each topographic image.

The height and peak force error channel images of PLA/PHB, uncross-linked and cross-linked PLA/PHB/NC-C films are shown in Figure 4. The surface of neat PLA/PHB-C film comprises of a continuous PLA phase (light colored in the topographic image and darker in peak force error image) and PHB domains, having an RMSR of 12 nm (Figure 4a). This observation is in good agreement with the SEM analysis and confirmed the poor interfacial compatibility between PLA and PHB.

After adding the DCP, the morphology of PLA/PHB blends has been changed (Figure 4b). The cross-linking agent reduced the size of PHB domains and the separation between the phases becomes gradual, resulting in better dispersion of PHB domains in PLA. Moreover, due to the DCP, the surface of PLA/PHB/D-C sample became rougher, RMSR increasing from 12 to 22 nm. This shows that PHB attached to PLA during reactive cross-linking and caused an increase of RMSR as compared with the uncross-linked PLA/PHB. The subsequent addition of NC in the PLA/PHB matrix resulted in a higher roughness (RMSR 32 nm). The freeze-dried NCs (marked with yellow arrows) are randomly distributed on the film surface (Figure 4c, Peak Force Error image). The PLA/PHB/NC/D-C cross-linked nanocomposite exhibited a more homogenous dispersion of NC in the PLA/PHB matrix, many individual NCs being visible at the surface of the sample (yellow arrows, Figure 4d, Peak Force Error image). NC fibers appeared covered with a polymer layer suggesting that the simultaneous addition of NC and DCP enhanced the interfacial adhesion and influenced the compatibility between PLA and PHB matrices and, possibly, their crystallinity. This nanocomposite showed the highest RMSR value of 65 nm. The AFM results sustained the previously SEM observations concerning the effect of DCP in increasing the compatibility between the two polymers and the dispersion of cellulose nanocrystals.

### 3.3. Fourier Transform Infrared Analysis

**Figure 5:**
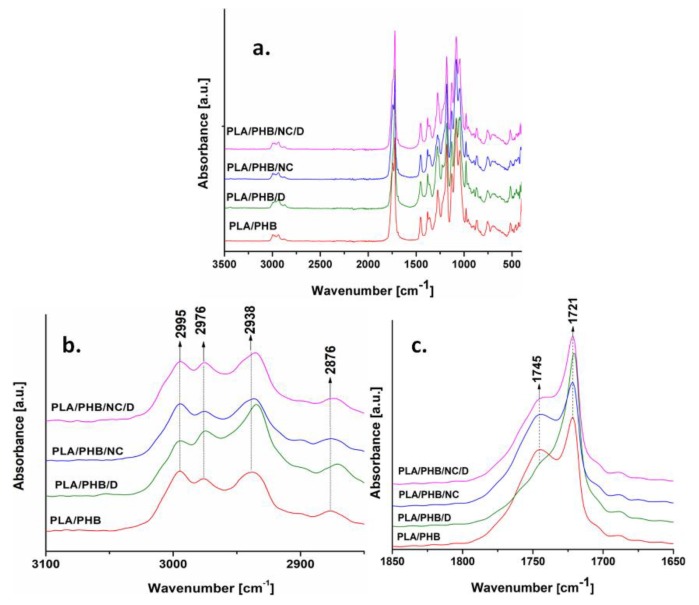
Full (**a**) and detailed (**b**,**c**) regions of FTIR spectra for PLA/PHB blends and nanocomposites films.

The FTIR−attenuated total reflectance (ATR) technique was used to identify the structural changes resulting from the chemical reactions induced by DCP in PLA/PHB/NC nanocomposites. The representative characteristic peaks of PLA and PHB polymers were noticed in both PLA/PHB blends and nanocomposites (Figure 5a) [7,13,43].

Several structural changes were noticed after cross-linking with DCP. Thus, the peak assigned to CH_3_ asymmetric stretching vibration at 2995 cm^−1^ changed relatively towards the peaks corresponding to –CH stretching vibrations at 2938 and 2876 cm^−1^, which were shifted to lower wavenumbers in the PLA/PHB and PLA/PHB/NC samples in the presence of DCP (Figure 5b). Moreover, the peak at 2938 cm^−1^, assigned to symmetric CH_3_ stretching, could also result from the decomposition of cumyloxy radicals into the acetophenone and methyl radicals [15].

Figure 5c shows that the shoulder at 1745 cm^−1^, assigned to the stretching vibration of C=O in amorphous lactides, becomes broader after the addition of NC and almost disappeared in the presence of DCP. This could indicate important changes in PLA crystallinity and some changes regarding the interactions between PLA and PHB after cross-linking. The broadening of this peak in PLA/PHB nanocomposites, especially in the presence of DCP, may arise from the molecular interaction between both polymers which has been ascribed to a transesterification reaction between PLA and PHB during melt reactive processing [44]. In order to confirm the grafting of PLA/PHB on the cellulose surface, the carbonyl index was calculated from the ratio between the absorbance of the carbonyl stretching vibration at 1721 and 1745 cm^−1^. Therefore, the carbonyl index of PLA/PHB composites increased from 1.27 to 2.15 in the presence of DCP, while for PLA/PHB/NC the same index varied from 1.2 to 1.87. This behavior suggests an increased crystallinity of the composites due to the cross-linking.

**Figure 6:**
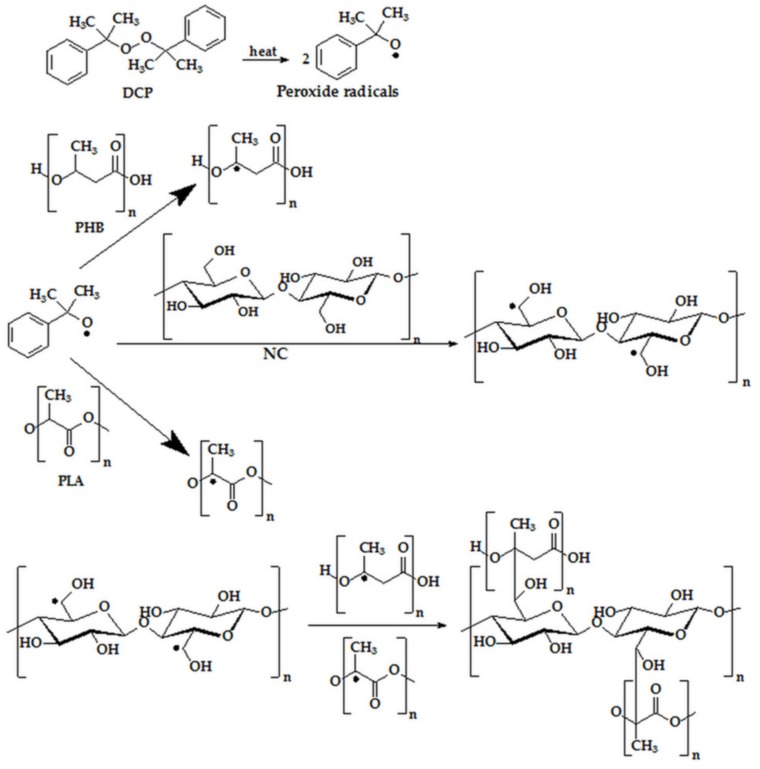
Mechanism of grafting PLA/PHB onto cellulose.

The general mechanism of peroxide radical initiated grafting of PLA or PHB onto cellulose comprises in the first step generation of the peroxide radicals followed by the formation of PLA/PHB radicals due to the preferentially attack on the tertiary carbon atoms, or in the case of cellulose, on the C6 position of the glucopyranose ring (Figure 6). In the propagation and termination steps, PLA/PHB and cellulose radicals react to each other, a demonstrated route through Electron Spin Resonance (ESR) measurements by other authors [45,46].

### 3.4. Differential Scanning Calorimetry

**Figure 7:**
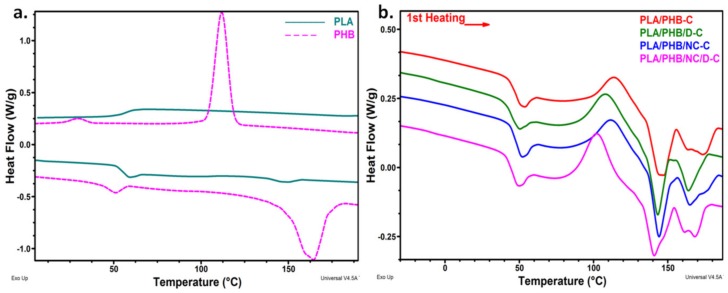
DSC curves of pristine PLA and PHB—first heating and cooling scans (**a**) and that of PLA/PHB blends and nanocomposites—first heating scan (**b**).

Figure 7 shows the first heating and cooling curves of neat PLA and PHB and the first heating curves of PLA/PHB blends and nanocomposites processed as films. The data measured from DSC curves were listed in Table 2. Pristine PLA and PHB samples have different glass transition temperatures (*T_g_*), the first at 56.7 °C and PHB at a much lower temperature (−10.0 °C) showing increased flexibility at room temperature. Although the melting peak of PHB (164.4 °C) is higher than that of PLA (149.5 °C), the melting range is still close for both polymers, between 130 °C and 160 °C for PLA and between 130 °C and 180 °C for PHB. This is important because it allows their blending in the melt state. Unlike PHB which is crystallized from the processing step, PLA shows a small exothermic crystallization peak during the first heating run at 120.8 °C and a very small crystallinity (Figure 7a, Table 2). Similar results were previously reported [7,10,47].

PHB/PLA blends and nanocomposites exhibit two glass transitions (Figure 7b) because PLA and PHB are not miscible. However, the *T*_g_ values are closer compared to that of pristine polymers (Table 2) suggesting compatibility between PLA and PHB. The smallest difference, of only 29 °C, between the *T*_g_ values of PLA and PHB, instead of 67 °C in the case of pristine polymers, was noticed for PLA/PHB/NC (Table 2). This shows the influence of NC in increasing the compatibility between PLA and PHB. Arrieta et al. [44] also studied PLA/PHB/NC nanocomposites and observed no *T_gPLA_* shift to lower temperature or compatibilising effect of NC. To the best of our knowledge this compatibilizing effect of nanocellulose in the PLA/PHB blend was experienced for the first time. It is worth mentioning that *T_gPHB_* shift was more important (17–20 °C) in uncross-linked samples—the addition of DCP decreased *T_gPLA_* with 10–12 °C, which may be caused by a compatibilising effect or a reduction of the molecular weight of PLA due to the chain scissions induced by DCP [19].

Both NC and DCP led to a shift of the cold crystallization to lower temperatures but the effect of DCP was more important (Table 2). It is well known that NC acts as a nucleating agent and induces faster crystallization to PLA [23,42,44]. However, the shift of *T_cc_* with 6 and 12 °C, observed in PLA/PHB/D and PLA/PHB/NC/D cross-linked samples, may be due to a nucleating effect of DCP or its decomposition derivatives or to the lower molecular weight products resulted from the cross-linking or decomposition processes [15,44]. The higher flexibility of PHB chains may also favor PLA crystallization, considering that all the blends and nanocomposites have a lower *T_cc_* value compared to PLA and a cold crystallization enthalpy about 15 times higher than that of PLA (Table 2). A higher increase of Δ*H_cc_* was noticed in the DCP cross-linked samples due to the presence of low-molecular-weight products with increased mobility, which favors PLA crystallization [15].

PLA/PHB blends and nanocomposites showed two main peaks during the melting process (Figure 7b), the first one due to the melting of “as formed” and recrystallized PLA (*T_mPLA_*) and the second one, split into two small peaks, corresponding to PHB crystallites. It is worth noting the higher PLA crystallinity (*X_cPLA_*) observed in all the blends and nanocomposites compared to PLA, showing the effect of PHB chains with higher flexibility. Further increased *X_cPLA_* values obtained in PLA/PHB/D, PLA/PHB/NC and PLA/PHB/NC/D confirm the effects of NC and DCP as nucleating agents.

No crystallization phenomena were observed for both blends and nanocomposites (Figure S1) in the cooling scan due to the slow crystallization of these polymers. DSC thermal parameters obtained for the second heating scan (Figure S2, Table S1) had the same variation observed in the first heating scan. Only a sharp endothermic peak appeared just before the glass transition of PLA, typically attributed to the enthalpy relaxation on heating mostly due to the previous physical aging of the polymer [7,26]. This peak was more pronounced for neat PLA/PHB and PLA/PHB/NC samples, probably because DCP led to lower molecular weight products with increased mobility.

In summary, the DSC results clearly show the influence of NC and DCP on the glass transition temperatures, crystallinity, and cold crystallization process. NC and DCP showed nucleating activity and favored recrystallization of PLA. Moreover, DCP led to lower molecular weight products with increased mobility and influenced the melting-recrystallization processes.

### 3.5. Thermogravimetric Analysis

Initial degradation temperature (*T_on_*), maximum degradation temperatures of PHB and PLA (*T_max1_* and*T_max2_*) were calculated from the first derivative of the TGA curves (DTG) while the temperature at 10% weight loss (*T_10%_*), weight loss corresponding to the temperature of 200 °C (*W_loss200_*), which is the highest temperature used in this study for the 3D printing, and the residue (*R_700_*) were calculated from TGA curves and the results are compiled in Table 3.

**Figure 8:**
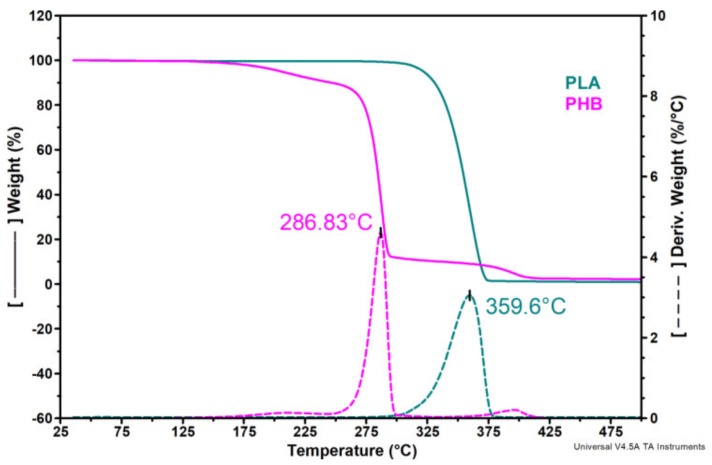
Thermogravimetric curves (TG) and their derivatives (DTG) for PLA and PHB.

Figure 8 shows the thermal behavior of the two pristine polymers. The first degradation peak in the derivative thermogravimetric (DTG) curves comes from the decomposition of the PHB component while the second one is due to hydrolysis and oxidative chain scission of PLA, as resulted from the comparison with the DTG profiles of pure components and literature [44,46]. A small shoulder was noticed in the DTG curves at about 195 °C. This is probably due to the release of TBC plasticizer from the PHB matrix as mentioned in a previous study on the same type of PHB [47] (Figure 8).

**Figure 9:**
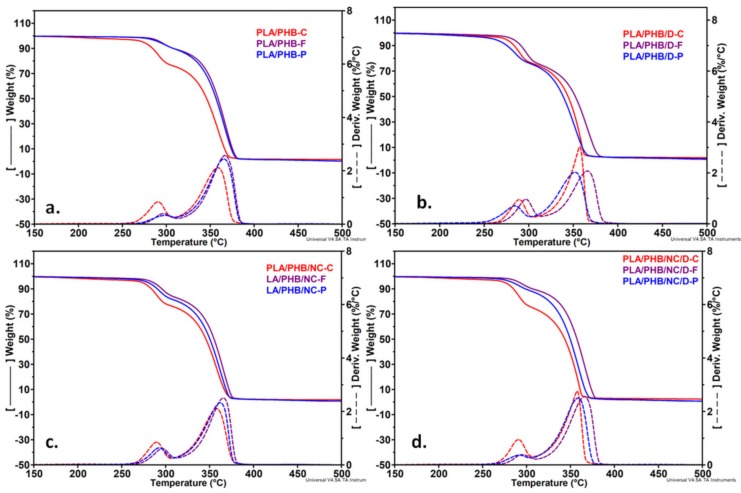
Thermogravimetric curves (TG) and their derivatives (DTG) for uncross-linked (**a**) and cross-linked (**b**) PLA/PHB blends and uncross-linked (**c**) and cross-linked (**d**) PLA/PHB nanocomposites films (C), filaments (F) and printed meshes (P).

All PLA/PHB blends and nanocomposites exhibited a two-step degradation profile regardless of the processing method (Figure 9a–d). PLA is more thermally stable than PHB and the blending of these two polymers resulted in an intermediary behavior, the *T_on_* of PLA75/PHB25 being located between those of individual polymers (Figure 9). This may be due to the hydrolysis reaction which is catalyzed by the carboxyl end groups of polyester resulted after thermal degradation [48].

All PLA/PHB blends and nanocomposites are thermally stable during the extrusion (170 °C) and the 3D printing process (200 °C) (Figure 9, Table 3). The poor thermal stability was noticed in the case of PLA/PHB/D-P. The degradation process occurred at a lower temperature (with 13–17 °C) for PLA/PHB/D-P compared to PLA/PHB/D film and filament, probably due to the DCP effect on the formation of low molecular weight compounds during 3D-printing and the degradation accelerated by the free radicals. Further, the addition of NC (PLA/PHB/NC/D-P), leads to the stabilization of PLA/PHB/D-P through the formation of an improved interface between PLA and PHB [44], also observed in the SEM images (Figure 3). Moreover, during reactive cross-linking, the sulphate groups of NC may be inactivated so delaying the degradation process.

An important increase of *T_10%_* values, even more than 10 °C, was observed for PLA/PHB and PLA/PHB nanocomposites after extrusion and 3D-printing compared with the same samples as films (Table 3). Still, this effect was more pronounced in the case of extruded filaments. A shift of both *T_max1_* and *T_max2_* was seen for the PLA/PHB and PLA/PHB/NC samples processed as filaments and printed meshes due to the orientation effect. This induced orientation is diminished in the presence of DCP, mainly for the 3D printed samples considering the higher temperature and shear forces involved in the printing process.

Notably, the weight loss determined at 200 °C and the residue at 700 °C presented lower values when the samples were processed as filaments and printed meshes as compared with the films. The compression-molded samples have a *W_loss200_* smaller than 2% while the extruded and 3D printed samples exhibit outstanding thermal stability as the values of *W_loss200_* are lower than 1% (Table 3). This behavior is of great importance considering that the usual processing temperatures of PLA/PHB are around 200 °C.

Overall, the samples processed as filaments showed the best thermal stability with the highest values for all the thermal parameters (Table 3). This may be related to the induced orientation during the extrusion process. During hot stretching, tensile forces may act upon the filament, resulting in a tension induced crystallization of PLA [49,50].

It can be concluded that the thermal stability of PLA/PHB blends and nanocomposites is not diminished after extrusion and 3D printing, thus ensuring a wide processing window and no risk of thermal degradation which is important for the industrial applications.

### 3.6. Dynamic Mechanical Analysis

**Figure 10:**
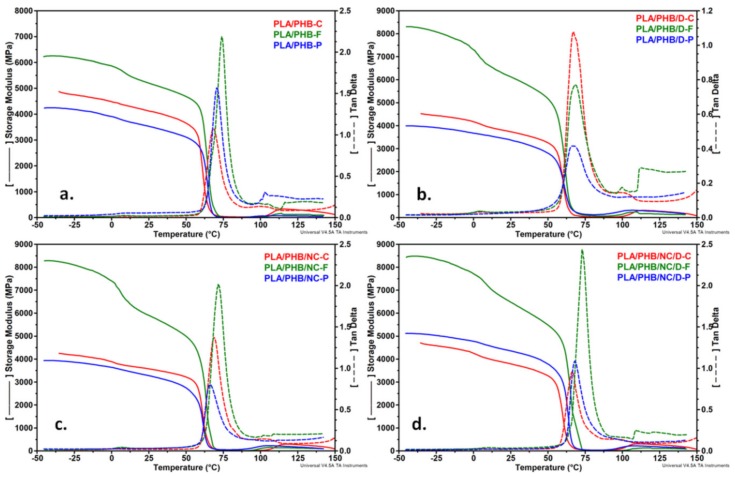
Storage modulus and tanδ plots for the PLA/PHB blends (**a**,**b**) and nanocomposites (**c**,**d**) as films, filaments and printed meshes.

Figure 10 shows the storage modulus (*E’*) and damping factor (tan*δ*) spectra of PLA/PHB blends and nanocomposites as films and after processing as filaments and printed meshes over a temperature range from −50 to 150°C.

The extrusion process led to an overall increase in the modulus of PLA/PHB-F blends and nanocomposite as compared with the one of samples processed as films in both glassy (−10 and 25 °C) and rubbery (95 °C) states (Table 4). This increase in modulus may be related to the chain orientation and increased crystallinity induced by the extrusion process [15]. Moreover, this effect is more pronounced in the case of PLA/PHB/NC/D-F as a result of increased interfacial adhesion of NC in the presence of DCP.

3D printed samples showed a distinct behavior, *E’* values decreased in the glassy state, probably due to a decrease in the molecular weight and formation of large fractions of low molecular weight chains which are less effective at storing energy under tensile load. Like the sample processed by extrusion, PLA/PHB/NC/D-P sample had higher *E’* values as a result of increased interfacial adhesion.

Rytlewski et al. considered that the higher values of the storage modulus recorded in the cold crystallization temperature range are an indication of a better crystallization ability of the samples containing DCP and can be considered an indirect measure of the cross-links formed between the PLA chains [15]. This observation is sustained by the crystallinity fractions of PLA calculated from DSC results (Table 2). The spectra of mechanical damping factor (tanδ) prior to and after extrusion or 3D printing processing revealed two relaxation peaks ascribed to the *T_g_* of both polymers (Figure 10, Table 4) as also noticed in the DSC curves. An increase of *T_g_* was seen for all the samples after extrusion and 3D-printing processing probably due to the increased crystallinity and reduced chain mobility in the amorphous regions. The increased *T_g_* was more pronounced in the case of filaments, in agreement with other reports [51]. An enhanced interfacial adhesion with the polymer matrix is possible to occur during processing through the formation of cross-links initiated by the peroxide in addition to the interactions of the ester groups [17]. As a result, polymer molecular motions are restricted, and the glass transition occurs at a higher temperature for both PHB and PLA. It is worth mention that the tanδ height decreased and the peak becomes broader for PLA/PHB/D-F and PLA/PHB/D-P samples compared to PLA/PHB/D-C, which indicates an increased stiffness due to the cross-linking process.

The nanocomposites processed as filaments showed the best thermo-mechanical properties due to the distinct morphology and better dispersion of NC also observed by SEM. This is due to the extrusion process in which the polymer chains rearrangement in regular lamellar crystals and interlamellar-non-crystalline regions along with the orientation direction is induced by extrusion.

This in-depth analysis of PLA/PHB/NC nanocomposites revealed that the thermo-mechanical properties of the nanocomposites filaments and printed meshes were not diminished upon extrusion and 3D-printing processing. Moreover, the addition of NC and reactive blending leads to notable positive changes in thermal stability and mechanical properties.

## 4. Conclusions

Cellulose nanocrystals were synthesized from plum seed shells by acid hydrolysis and were successfully incorporated in the PLA/PHB matrix by a reactive blending method. The use of a cross-linking agent improved both the dispersion of NC in the nanocomposites and the interfacial adhesion between PLA and PHB components as shown from SEM and AFM results. The best thermal stability and highest maximum degradation temperature were obtained for DCP treated nanomaterials, especially in the case of PLA/PHB/NC nanocomposite filaments as compared with 3D printed meshes and films. DSC analysis showed that the addition of both NC and DCP favors the PLA recrystallization, the cold crystallization peak being shifted to lower temperatures. An overall increase in the modulus of PLA/PHB nanocomposite filaments as compared with the PLA/PHB composite films in both glassy (−10 and 25 °C) and rubbery (95 °C) state was observed from DMA analysis in relation with the chain orientation and increased crystallinity. Furthermore, above glass transition temperature the nanocomposites filaments and printed meshes exhibited higher values of E’ which confirm the formation of cross-linked structures and better interfacial adhesion with the polymer matrix especially in the presence of DCP. The cumulated effects represented by the improved thermal stability, enhanced interfacial adhesion between PLA and PHB, improved dispersion of NC in PLA/PHB matrix on the final properties of PLA/PHB/NC nanocomposites filaments and 3D printed meshes demonstrate that these nanomaterials meet the high standards of engineering applications provided the right processing method is chosen.

## Figures and Tables

**Table 1:** Nanocomposites formulations and notation after different processing methods.

Sample Code			Composition (wt.%)			
Compression	Extrusion	3D Printing	PLA	PHB	NC	DCP
PLA/PHB-C	PLA/PHB-F	PLA/PHB-P	75	25	-	-
PLA/PHB/D-C	PLA/PHB/D-F	PLA/PHB/D-P	74.25	24.75	-	1
PLA/PHB/NC-C	PLA/PHB/NC-F	PLA/PHB/NC-P	74.25	24.75	1	-
PLA/PHB/NC/D-C	PLA/PHB/NC/D-F	PLA/PHB/NC/D-P	73.50	24.50	1	1

**Table 2:** DSC data corresponding to the first heating scan for PLA/PHB blends and nanocomposites.

Sample	*T_gPHB_* (°C)	*T_gPLA_* (°C)	*T_cc_* (°C)	Δ*H_cc_* (J/g)	*T_mPLA_* (°C)	Δ*H_mPLA_* (J/g)	*T_mPHB1_/T_mPHB2_* (°C)	Δ*H_mPHB1_/*Δ*H_mPHB2_* (J/g)	X_c_ (%)	
									*X_cPLA_*	*X_cPHB_*
PLA	-	56.7	120.8	1.31	149.5	2.11	-	-	0.9	0
PHB	−10.0	-	-	-	-	-	161.0/164.4	27.2/30.8	0	39.8
PLA/PHB-C	6.7	48.6	114.2	17.0	144.7	16.0	163.5/173.7	4.6/7.8	16.4	34.0
PLA/PHB/D-C	−8.7	44.5	108.5	22.0	143.2	15.3	163.7/-	11.17/-	37.9	30.6
PLA/PHB/NC-C	19.8	49.2	112.1	19.7	144.1	16.2	164.8/172.0	8.2/3.4	40.1	31.9
PLA/PHB/NC/D-C	−7.6	46.5	102.3	23.8	140.8	18.0	161.3/168.1	5.0/6.6	42.6	31.9

**Table 3:** TG/DTG data corresponding to PLA/PHB blends and nanocomposites.

Sample	PLA/PHB			PLA/PHB/D			PLA/PHB/NC			PLA/PHB/NC/D		
Processing Form	C	F	P	C	F	P	C	F	P	C	F	P
*T_on_*, °C	267	277	272	268	272	255	269	273	271	270	276	267
*T_10%_,* °C	284	308	308	284	291	275	284	296	290	285	307	299
*W_loss200_*, %	1.34	0.53	0.55	1.48	0.81	1.48	1.64	0.79	0.91	1.32	0.56	0.66
*T_max1_*,°C	290	296	297	289	296	284	289	296	292	291	294	291
*T_max2_*, °C	359	367	366	357	366	352	358	366	362	358	366	359
*R_700°C_*, %	1.21	0.22	0.21	1.42	0.46	0.61	1.45	0.61	0.66	1.92	0.27	0.44

**Table 4:** Values of *E’* in glassy and rubbery state and the glass transition temperature determined from tanδ curves (*T_gPHB_* and *T_gPLA_*).

Sample	*T_gPHB_* (°C)	*T_gPLA_* (°C)	*E’* (MPa) −10 °C	*E’* (MPa) 25 °C	*E’* (MPa) 95°C
PLA/PHB-C	3.4	67.1	4934	4319	19
PLA/PHB-F	9.0	73.9	6004	5202	6
PLA/PHB-P	9.3	70.72	4047	3532	39
PLA/PHB/D-C	5.4	66.9	4312	3734	32
PLA/PHB/D-F	4.2	68.2	7724	6126	91
PLA/PHB/D-P	-	66.0	3789	3375	214
PLA/PHB/NC-C	1.8	68.8	4033	3574	18
PLA/PHB/NC-F	6.7	71.9	7773	5903	35
PLA/PHB/NC-P	6.3	66.0	3742	3280	133
PLA/PHB/NC/D-C	3.2	66.5	4431	3792	48
PLA/PHB/NC/D-F	9.2	72.9	8025	6482	30
PLA/PHB/NC/D-P	7.0	68.0	4884	4358	89

## Supplementary Materials

The following are available online at https://www.mdpi.com/2079-4991/10/1/51/s1, Figure S1: DSC curves of PLA/PHB blends and nanocomposites - first cooling scan, Figure S2: DSC curves of PLA/PHB blends and nanocomposites - second heating, Table S1: DSC data corresponding to the second heating scan.
