# Supplementary material for: Morpho-Structural, Thermal and Mechanical Properties of PLA/PHB/Cellulose Biodegradable Nanocomposites Obtained by Compression Molding, Extrusion, and 3D Printing
Source: Nanomaterials (Basel). 2019 Dec 24;10(1):51. doi: 10.3390/nano10010051 (PMC7023130; doi:10.3390/nano10010051)
Supplement: Supplementary file 1 [file nanomaterials-10-00051-s001.pdf]

## Supplementary Materials

# Morpho-Structural, Thermal and Mechanical Properties of PLA/PHB/Cellulose Biodegradable Nanocomposites Obtained by Compression Molding, Extrusion, and 3D Printing

**Adriana Nicoleta Frone[1,*], Dan Batalu[2], Ioana Chiulan[1], Madalina Oprea[1], Augusta Raluca Gabor[1], Cristian Andi Nicolae[1], Valentin Raditoiu[1], Roxana Trusca[3] and Denis Mihaela Panaitescu[1]**

[1] National Institute for Research & Development in Chemistry and Petrochemistry ICECHIM, Polymer Department, 202 Splaiul Independentei, 060021, Bucharest, Romania; ciucu_adriana@yahoo.com (A.F.); ioana.chiulan@icechim.ro (I.C.); madalinna_09@yahoo.com (M.O.); ralucagabor@yahoo.com (R.G.); ca_nicolae@yahoo.com (C.N.); vraditoiu@yahoo.com (V.R.); panaitescu@icechim.ro (D.P)

[2] Materials Science and Engineering Faculty, University Politehnica of Bucharest, 060042, Bucharest, Romania; dan_batalu@yahoo.com (D.B.)

[3] Science and Engineering of Oxide Materials and Nanomaterials, University Politehnica of Bucharest, 1-7 Gh. Polizu Street, 011061 Bucharest, Romania; truscaroxana@yahoo.com (R.T.)

* Correspondence: ciucu_adriana@yahoo.com; Tel.: +40-213163068

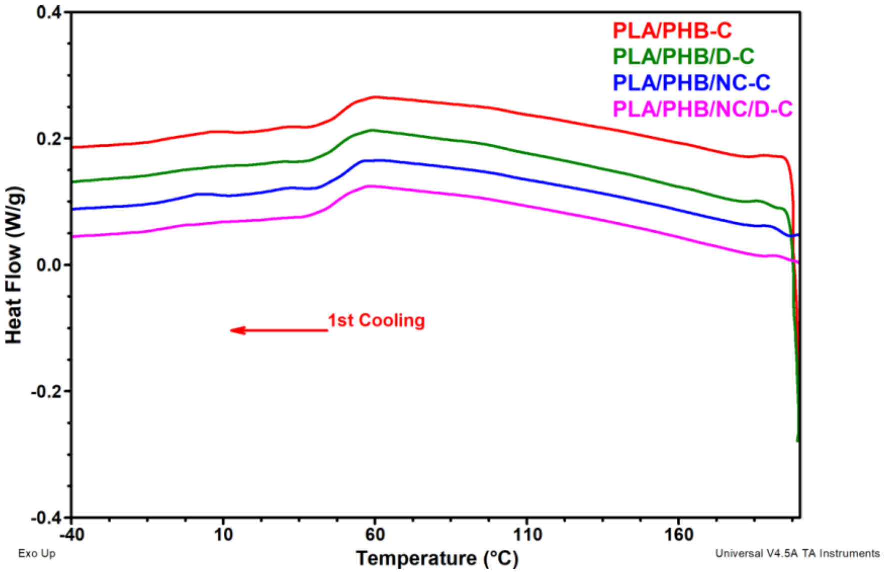


**Figure S1.** DSC curves of PLA/PHB blends and nanocomposites - first cooling scan

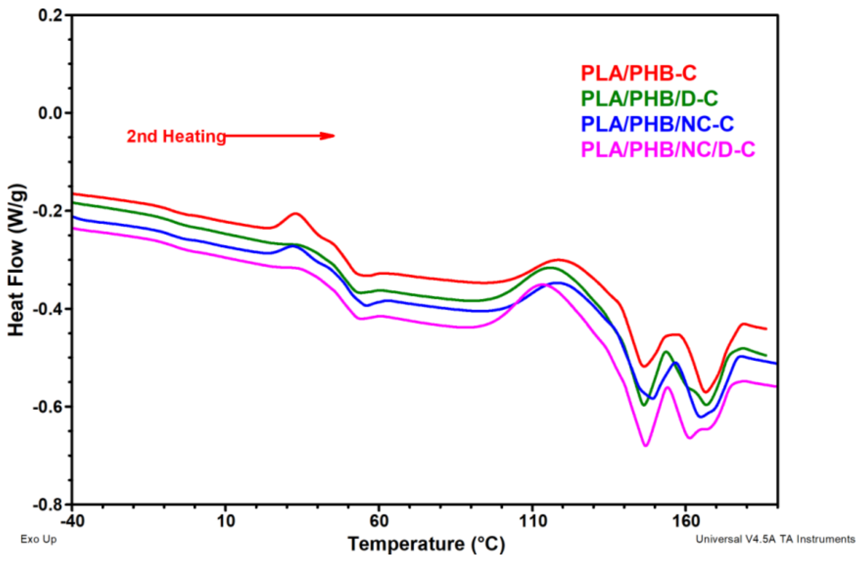


**Figure S2.** DSC curves of PLA/PHB blends and nanocomposites - second heating

**Table S1.** DSC data corresponding to the second heating scan

| **Sample** | $T_{gPHB}$ (°C) | $T_{gPLA}$ (°C) | $T_{cc}$ (°C) | $\Delta H_{cc}$ (J/g) | $T_{mPLA}$ (°C) | $\Delta H_{mPLA}$ (J/g) | $T_{mPHB1}$/ $T_{mPHB2}$ (°C) | $\Delta H_{mPHB1}/\Delta H_{mPHB2}$ (J/g) |
|---|---|---|---|---|---|---|---|---|
| PLA | - | 58.0 | 123.3 | 1.0 | 150.5 | 1.90 | - | - |
| PHB | -10.0 | - | - | - | - | - | 158.4/165.3 | 51.5/12.2 |
| PLA/PHB-C | -7.7 | 50.5 | 120.0 | 7.2 | 146.4 | 9.5 | 166.5 | 11.5 |
| PLA/PHB/D-C | -6.9 | 47.6 | 116.5 | 10.3 | 146.3 | 10.3 | 163.7/166.7 | 4.5/6.9 |
| PLA/PHB/NC-C | -8.6 | 49.8 | 119.5 | 9.4 | 149.1 | 9.5 | 164.8 | 11.1 |
| PLA/PHB/NC/D-C | -6.4 | 47.5 | 114.1 | 11.1 | 146.3 | 11.9 | 163.1/166.6 | 6.8/4.7 |
